# Pedigree relationships to control inbreeding in optimum-contribution selection realise more genetic gain than genomic relationships

**DOI:** 10.1186/s12711-019-0475-5

**Published:** 2019-07-08

**Authors:** Mark Henryon, Huiming Liu, Peer Berg, Guosheng Su, Hanne Marie Nielsen, Gebreyohans T. Gebregiwergis, A. Christian Sørensen

**Affiliations:** 1 Danish Pig Research Centre, SEGES, Axeltorv 3, 1609 Copenhagen V, Denmark; 2 School of Agriculture and Environment, University of Western Australia, 35 Stirling Highway, Crawley, WA 6009 Australia; 3 Institute for Molecular Biology and Genetics, Aarhus University, P.O. Box 50, 8830 Tjele, Denmark; 4 Department of Animal and Aquaculture Sciences, Norwegian University of Life Sciences, Ås, Norway

## Abstract

**Background:**

We tested the premise that optimum-contribution selection with pedigree relationships to control inbreeding (POCS) realises at least as much true genetic gain as optimum-contribution selection with genomic relationships (GOCS) at the same rate of true inbreeding.

**Methods:**

We used stochastic simulation to estimate rates of true genetic gain realised by POCS and GOCS at a 0.01 rate of true inbreeding in three breeding schemes with best linear unbiased predictions of breeding values based on pedigree (PBLUP) and genomic (GBLUP) information. The three breeding schemes differed in number of matings and litter size. Selection was for a single trait with a heritability of 0.2. The trait was controlled by 7702 biallelic quantitative-trait loci (QTL) that were distributed across a 30-M genome. The genome contained 54,218 biallelic markers that were used in GOCS and GBLUP. A total of 6012 identity-by-descent loci were placed across the genome in base populations. Unique alleles at these loci were used to calculate rates of true inbreeding. Breeding schemes were run for 10 discrete generations. Selection candidates were genotyped and phenotyped before selection.

**Results:**

POCS realised more true genetic gain than GOCS at a 0.01 rate of true inbreeding in all combinations of breeding scheme and prediction method. POCS realised 14 to 33% more true genetic gain than GOCS with PBLUP in the three breeding schemes. It realised 1.5 to 5.7% more true genetic gain than GOCS with GBLUP.

**Conclusions:**

POCS realised more true genetic gain than GOCS because it managed expected genetic drift without restricting selection at QTL. By contrast, GOCS penalised changes in allele frequencies at markers that were generated by genetic drift and selection. Because these marker alleles were in linkage disequilibrium with QTL alleles, GOCS restricted changes in allele frequencies at QTL. This provides little incentive to use GOCS and highlights that we have more to learn before we can control inbreeding using genomic relationships in selective-breeding schemes. Until we can do so, POCS remains a worthy method of optimum-contribution selection because it realises more true genetic gain than GOCS at the same rate of true inbreeding.

## Background

**Fig. 1:**
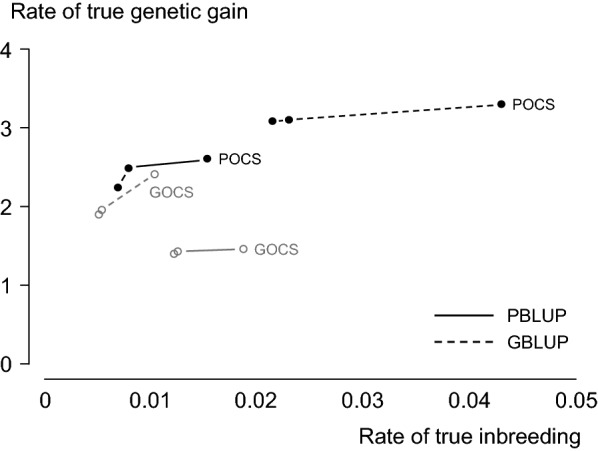
Rate of true genetic gain realised by POCS and GOCS plotted against ${\Delta \text{F}}_{\text{true}}$ in simulated breeding schemes with two prediction methods (PBLUP and GBLUP). Adapted from Table 2 in Sonesson et al. [4]

The aim of most animal-breeding schemes is to maximise rates of true genetic gain ($\Delta {\text{G}}_{\text{true}}$) at acceptable rates of true inbreeding ($\Delta {\text{F}}_{\text{true}}$). $\Delta {\text{G}}_{\text{true}}$ is calculated as the increase in true breeding value (TBV) averaged across animals in a breeding population. $\Delta {\text{F}}_{\text{true}}$ is calculated from the average true inbreeding coefficient of the animals, where the true inbreeding coefficient of an individual is the proportion of loci in its genome with alleles that are identical-by-descent (IBD). Both $\Delta {\text{G}}_{\text{true}}$ and $\Delta {\text{F}}_{\text{true}}$ are unobservable in practice. They need to be predicted. The best selection method to use these predictions and fulfil the aim of most animal-breeding schemes is optimum-contribution selection (OCS). OCS maximises rates of predicted genetic gain while controlling inbreeding at given rates of predicted inbreeding [1, 2]. It does this by optimising the genetic contribution of each selection candidate to the next generation. One of the benefits of OCS is that it can optimise genetic contributions when different sources of information are used to predict $\Delta {\text{G}}_{\text{true}}$ and control $\Delta {\text{F}}_{\text{true}}$ [3]. $\Delta {\text{G}}_{\text{true}}$ is, more often than not, predicted using best linear unbiased prediction (BLUP) of breeding values based on pedigree or genomic information, hereafter referred to as PBLUP and GBLUP. $\Delta {\text{F}}_{\text{true}}$ is predicted and controlled using pedigree or genomic relationships, hereafter referred to as OCS with pedigree (POCS) or genomic relationships (GOCS). GOCS became the method-of-choice for OCS with GBLUP when Sonesson et al. [4] used stochastic simulation to recommend that the information used to predict $\Delta {\text{G}}_{\text{true}}$ should also be used to predict and control $\Delta {\text{F}}_{\text{true}}$. Their reasoning was that GOCS predicted and controlled $\Delta {\text{F}}_{\text{true}}$ more accurately when it was used with GBLUP, while POCS predicted and controlled $\Delta {\text{F}}_{\text{true}}$ more accurately with PBLUP. However, this reasoning did not consider $\Delta {\text{G}}_{\text{true}}$. When we plotted $\Delta {\text{G}}_{\text{true}}$ realised by Sonesson et al. [4] against $\Delta {\text{F}}_{\text{true}}$, we saw that POCS realised more $\Delta {\text{G}}_{\text{true}}$ than GOCS, even at similar $\Delta {\text{F}}_{\text{true}}$ (Fig. 1). We are generally supported by Clark et al. [5], who found that, with few exceptions, POCS realised just as much $\Delta {\text{G}}_{\text{true}}$ as GOCS with both PBLUP and GBLUP, despite being compared at the same rates of genomic inbreeding. Comparing POCS and GOCS at the same rates of genomic inbreeding, rather than $\Delta {\text{F}}_{\text{true}}$, would have favoured GOCS, given that GOCS maximises rates of predicted genetic gain while controlling rates of genomic inbreeding. Our interpretation of these studies led us to believe that POCS realises at least as much ${\Delta \text{G}}_{\text{true}}$ as GOCS at the same ${\Delta \text{F}}_{\text{true}}$. We tested this premise by stochastic simulation. We compared ${\Delta \text{G}}_{\text{true}}$ realised by POCS and GOCS at ${\Delta \text{F}}_{\text{true}}$ = 0.01 $\left({0.01 {\Delta \text{F}}_{\text{true}}} \right)$ in three breeding schemes with PBLUP and GBLUP. We also simulated OCS with IBD relationships (IOCS) and replaced predictions of breeding values with TBV as points of reference. Results that highlight the mechanisms underlying POCS and GOCS are presented.

## Methods

### Design

We used stochastic simulation to estimate ${\Delta \text{G}}_{\text{true}}$ realised by POCS and GOCS at $0.01 {\Delta \text{F}}_{\text{true}}$ in three breeding schemes with PBLUP and GBLUP. Put simply, we calibrated POCS and GOCS to realise $0.01 {\Delta \text{F}}_{\text{true}}$ and compared their ${\Delta \text{G}}_{\text{true}}$. We also simulated IOCS—OCS with IBD relationships—and replaced predictions of breeding values with TBV as points of reference. Selection was for a single trait that had a heritability of 0.2 and was controlled by 7702 biallelic quantitative-trait loci (QTL). The QTL were randomly distributed across a 30-M genome that consisted of 18 pairs of autosomal chromosomes. Each chromosome was 167 cM long. The genome contained 54,218 biallelic markers that were used in GOCS and GBLUP. These markers were randomly distributed across the genome and in linkage disequilibrium (LD) with the QTL. A total of 6012 IBD loci were placed evenly across the genomes of animals in base populations. Unique alleles at these loci were used to calculate ${\Delta \text{F}}_{\text{true}}$ and carry out IOCS. The number of chromosomes and LD between alleles at the markers were simulated to resemble those in three commercial breeds of Danish pigs [6]. Breeding schemes were run for 10 discrete generations ($t$ = 1 … 10). Animals in the base populations were randomly selected in generation $t$ = 1. In generations $t$ = 2 … 10, selection candidates were allocated matings by OCS. All animals were genotyped before selection; all candidates in generations $t$ = 2 … 10 were phenotyped for the trait under selection. Each combination of OCS method, breeding scheme, and prediction method was replicated 200 times. We present ${\Delta \text{G}}_{\text{true}}$ realised at $0.01 {\Delta \text{F}}_{\text{true}}$ in generations $t$ = 4 … 10 and results that highlight the mechanisms underlying POCS and GOCS.

### Breeding schemes

The three breeding schemes differed in number of matings and litter size.

#### M25L5

Twenty-five matings were allocated to 125 selection candidates by OCS in generations $t$ = 2 … 10. There was no upper limit for the number of matings that were allocated to each male; males were allocated 0, 1, 2 … or 25 matings. Twenty-five females were allocated a single mating. The 25 sire and dam matings were paired randomly. Each pair (dam) produced five offspring, resulting in 25 full-sib families and 125 offspring. Offspring were assigned as males or females with a probability of 0.5.

#### M25L20

This scheme is as for breeding scheme M25L5 with two exceptions. First, 25 matings were allocated to 500 candidates. Second, each dam produced 20 offspring, resulting in 25 full-sib families and 500 offspring.

#### M100L5

This scheme is as for breeding scheme M25L5 with two exceptions. First, 100 matings were allocated to 500 candidates. Males were allocated 0, 1, 2 … or 100 matings and 100 females were allocated a single mating. Second, each dam produced five offspring, resulting in 100 full-sib families and 500 offspring.

### Simulation procedure

#### Generations − 1000 to − 1: founder population

LD between the 54,218 markers and 7702 QTL was established in a founder population using a Fisher-Wright inheritance model [7, 8]. The founder population was simulated for 1000 discrete generations ($t$ = − 1000 … − 1) with 25 males and 25 females, and an effective-population size of 50, in each generation. In generation $t$ = − 1, the founder population was in recombination-drift-mutation-selection equilibrium. We considered the founder population to be in equilibrium when the numbers of segregating markers and QTL, the level of heterozygosity averaged over all segregating markers and QTL, and the average LD between segregating markers that were 0.25, 0.5, 1, 2, 5, and 10 cM apart became constant across generations.

The founder population was initiated with 25 males and 25 females in generation $t$ = − 1000. Their 30-M genomes consisted of 3 × 10^7^ monomorphic loci with wild-type alleles that were placed evenly across the genome at 10^4^ loci per cM. Every eighth locus harboured a QTL that controlled the trait under selection. The remaining loci were markers.

The males and females in subsequent generations were simulated by randomly sampling a sire and dam with replacement from the 25 males and 25 females in the previous generation. Bi-allelic polymorphism at each locus was generated with a mutation rate of 4 × 10^−6^ per locus using an infinite-sites mutation model [9]. An additive-genetic effect for the mutant allele at each QTL was sampled from an exponential distribution. The sign of each additive-genetic effect was negative with a probability of 0.9. The additive-genetic effects of the wild-type alleles were zero. Selection was introduced by sampling 25 males and 25 females that were above a 5% percentile for TBV. The TBV of the $i$th animal in the founder population, ${\text{a}}_{i}$, was calculated as ${\text{a}}_{i} = \mathop \sum \nolimits_{j = 1}^{{n_{\text{QTL}}}} {\text{x}}_{ij} {\text{g}}_{j}$, where $n_{\text{QTL}}$ = 3.75 × 10^6^ is the number of QTL across the genome, ${\text{x}}_{ij}$ is the number of copies of the mutant allele that animal $i$ inherited at the $j$th QTL (${\text{x}}_{ij}$ = 0, 1, 2), and ${\text{g}}_{j}$ is the additive-genetic effect of the mutant allele at the $j$th QTL. We introduced selection because animal populations are always under selection, which influences LD between alleles.

The 54,218 markers and 7702 QTL in our three breeding schemes were all segregating in generation $t$ = − 1 of the founder population. The additive-genetic effects of the mutant alleles at the 7702 segregating QTL were standardised so that the total additive-genetic variance for the trait under selection was equal to 1.0. No new mutations were generated after the founder population was simulated.

Chromosomes from the 50 animals in generation $t$ = − 1 of the founder population were pooled: 18 pools of 100 chromosomes. Each pool consisted of 50 chromosome pairs of the $i$th chromosome ($i$ = 1 … 18) from 50 founder animals. The breeding schemes were initiated by sampling base populations from these chromosome pools.

#### Generation 0: base populations

Each replicate combination of OCS method, breeding scheme, and prediction method was initiated by sampling a unique base population. Twenty-six males and 25 females were sampled in breeding schemes M25L5 and M25L20. Eleven males and 100 females were sampled in breeding scheme M100L5. The genotype of each base animal was sampled from the 18 pools of chromosomes in generation $t$ = − 1 of the founder population. For chromosome $i$ ($i$ = 1 … 18), two chromosomes were randomly sampled without replacement from the $i$th pool of 100 chromosomes. The sampled chromosomes were replaced before the next base animal was sampled. Base animals were assumed to be unrelated and non-inbred based on pedigree and IBD alleles. They were genotyped, but not phenotyped for the trait under selection.

#### Generation 1: random selection in base populations

Animals in the base populations were selected in generation $t$ = 1 by randomly culling a single male. In breeding schemes M25L5 and M25L20, 25 sires and 25 dams were selected. Each sire was mated with one dam. Each dam produced five offspring in breeding scheme M25L5 and 20 offspring in breeding scheme M25L20. In breeding scheme M100L5, 10 sires and 100 dams were selected. Each sire was mated with 10 dams and each dam produced five offspring. Randomly culling a single male enabled us to construct genomic-relationship matrices that were positive-definite. This is explained in more detail in section ‘Genomic and IBD-relationship matrices’.

#### Generations 2–10: optimum-contribution selection

Animals were selected and allocated matings by OCS in generations $t$ = 2 … 10. The phenotype of animal $i$, ${\text{p}}_{i}$, was calculated as ${\text{p}}_{i} = {\text{a}}_{i} + {\text{e}}_{i}$, where ${\text{a}}_{i}$ is the animal’s TBV and ${\text{e}}_{i}$ is its residual value. The TBV of animal $i$ was calculated as described for the founder population using the standardised additive-genetic effects of the mutant alleles at the 7702 QTL. Its residual value, ${\text{e}}_{i}$, was sampled from ${\text{e}}_{i} \sim N\left({0, \sigma_{e}^{2} = 4} \right)$.

### IBD loci

The 6012 IBD loci used to calculate ${\Delta \text{F}}_{\text{true}}$ and carry out IOCS were placed evenly across the genome of animals in the base populations at two IBD loci per cM (334 loci per chromosome). Each base animal was assigned two unique alleles at each IBD locus. IBD alleles could be traced back from each descendant to the base animal from which it was derived. A descendant was IBD at an IBD locus when it inherited two copies of a unique allele (i.e., both alleles at the IBD locus descended from the same unique allele in the base population). IBD loci were not involved in prediction.

### Optimum-contribution selection

POCS allocated matings to selection candidates in generations $t$ = 2 … 10 conditional on predicted breeding values and pedigree relationships. It did this by maximising a quadratic function, ${\text{U}}_{t}$, with respect to ${\mathbf{c}}$:

1 $${\text{U}}_{t} \left({\mathbf{c}} \right) = {\mathbf{c}}^{\prime}{\mathbf{g}} - \omega {\mathbf{c}}^{\prime}{\mathbf{Ac}},$$

where ${\mathbf{c}}$ is an $n$ vector of genetic contributions to the next generation and the number of matings allocated to each candidate is a linear function of these contributions, $n$ is the number of animals in the population traced back from the candidates in generation $t$ to the base population, ${\mathbf{g}}$ is an $n$ vector of PBLUP, GBLUP, or TBV, $\omega$ is a penalty applied to the expected average relationship of the next generation, and ${\mathbf{A}}$ is an $n \times n$ pedigree-relationship matrix. Elements of ${\mathbf{c}}$ were constrained to $0 \le {\text{c}}_{i} \le 0.5$ ($i = 1 \ldots n$) with ${\text{c}}_{i} = 0$ for animals that were not candidates for selection in generation $t$. Using these definitions, ${\mathbf{c^{\prime}g}}$ is the expected breeding value and ${\mathbf{c}}^{\prime}{\mathbf{Ac}}$ is the expected average relationship of the next generation. The penalty, $\omega$, was constant across generations. It was calibrated to realise $0.01 {\Delta \text{F}}_{\text{true}}$. We calibrated it by simulating 200 replicates of POCS in each combination of breeding scheme and prediction method with an initial $\omega$ and calculating the mean ${\Delta \text{F}}_{\text{true}}$ across the replicates. This process was repeated using different $\omega$ until the mean ${\Delta \text{F}}_{\text{true}}$ deviated from 0.01 by less than 0.0001. GOCS was carried out by replacing ${\mathbf{A}}$ with an $n \times n$ genomic-relationship matrix, ${\mathbf{G}}$. IOCS was carried out by replacing ${\mathbf{A}}$ with an $n \times n$ IBD-relationship matrix, ${\mathbf{B}}$. The method of POCS is described in full by Henryon et al. [10].

### Predicted breeding values

PBLUP for the trait under selection were estimated in generations $t$ = 2 … 10 by fitting an animal model to the phenotypes observed in generations 2 to $t$. The model was:

$${\mathbf{y}} = {\mathbf{Xb}} + {\mathbf{Za}} + {\mathbf{e}},$$

where ${\mathbf{y}}$ is an $n$ vector of phenotypes, ${\mathbf{b}}$ is an $h$ vector of fixed generation effects, $h$ is the number of generations with phenotypes, ${\mathbf{a}}$ is an $n$ vector of random animal effects, ${\mathbf{e}}$ is an $n$ vector of residual errors, and ${\mathbf{X}}$ and ${\mathbf{Z}}$ are incidence matrices. The (co)variance structure was:

$$\left({\begin{array}{*{20}c} {\mathbf{a}} \\ {\mathbf{e}} \\ \end{array}} \right)\sim N\left({\left[{\begin{array}{*{20}c} \mathbf{0} \\ \mathbf{0} \\ \end{array}} \right],\left[{\begin{array}{*{20}c} {{\mathbf{A}}\sigma_{a}^{2}} & \mathbf{0} \\ \mathbf{0} & {{\mathbf{I}}\sigma_{e}^{2}} \\ \end{array}} \right]} \right),$$

where ${\mathbf{I}}$ is an identity matrix, $\sigma_{a}^{2} = 1$ is the additive-genetic variance in generation $t$ = − 1 of the founder population, and $\sigma_{e}^{2} = 4$ is the residual variance that was used to sample phenotypes. GBLUP were estimated by replacing ${\mathbf{A}}$ with the genomic-relationship matrix, ${\mathbf{G}}$.

### Genomic and IBD-relationship matrices

#### Genomic-relationship matrices

Genomic-relationship matrices used in GOCS and GBLUP were constructed as ${\mathbf{G}} = {\mathbf{ZZ}}^{\prime}/{\text{s}}$, where ${\mathbf{G}}$ is an $n \times n$ matrix of genomic relationships, ${\mathbf{Z}} = {\mathbf{M}} - 1\left({2{\mathbf{p}}} \right)^{\prime}$, ${\mathbf{M}}$ is an $n \times m$ matrix of counts of the mutant allele for the $n$ animals at the $m$ = 54,218 markers with element ${\text{M}}_{ij} =$ 0, 1, or 2 for animal $i$ at marker $j$ ($i = 1 \ldots n$, *j* $= 1 \ldots m$), $1$ is an $n$ vector of ones, ${\mathbf{p}} = \left({{\text{p}}_{1}, {\text{p}}_{2}, \ldots {\text{p}}_{m}} \right)$ is an $m$ vector with ${\text{p}}_{j}$ the frequency of the mutant allele at marker $j$ in the base populations, and ${\text{s}} = 2{\mathbf{p}}^{\prime}\left({1 - {\mathbf{p}}} \right)$ transforms ${\mathbf{G}}$ towards the same scale as a pedigree-relationship matrix (adapted from VanRaden [11]).

We carried out two additional steps to ensure that ${\mathbf{G}}$ was positive-definite. First, the allele frequencies in ${\mathbf{p}}$ were calculated using all animals in the base populations, including the single male that was culled in generation $t = 1$ of each breeding scheme. Second, all base animals and selection candidates, except for the culled male, were included in ${\mathbf{G}}$. These steps generated linear independence in ${\mathbf{G}}$ because allele frequencies in ${\mathbf{p}}$ were calculated using an animal that was not included in ${\mathbf{G}}$.

#### IBD-relationship matrices

The IBD-relationship matrix, $${\mathbf{B}}$$, used in IOCS was an $$n \times n$$ matrix constructed as $${\text{B}}_{ij} = \frac{1}{{n_{\text{IBD}}}}\mathop \sum \nolimits_{k = 1}^{{n_{\text{IBD}}}} \left[{{\raise0.7ex\hbox{$1$} \!\mathord{\left/{\vphantom {1 2}}\right.\kern-0pt} \!\lower0.7ex\hbox{$2$}}\mathop \sum \nolimits_{u = 1}^{2} \mathop \sum \nolimits_{v = 1}^{2} \delta_{uv}} \right]$$, where element $${\text{B}}_{ij}$$ is the IBD relationship between animals $$i$$ and $$j$$ ($$i = 1 \ldots n$$, $$j = 1 \ldots n$$), $$n_{\text{IBD}}$$ = 6012 is the number of IBD loci, and $$\delta_{uv}$$ is the allele-sharing status. $$\delta_{uv}$$ was equal to 1 if allele $$u$$ of animal $$i$$ was identical to allele $$v$$ of animal $$j$$ at IBD locus $$k$$, and 0 otherwise.

### Rates of true genetic gain and true inbreeding

We present ${\Delta \text{G}}_{\text{true}}$ realised by POCS, GOCS, and IOCS at $0.01 {\Delta \text{F}}_{\text{true}}$ in each combination of breeding scheme and prediction method. ${\Delta \text{G}}_{\text{true}}$ and ${\Delta \text{F}}_{\text{true}}$ are presented as means (± SD) of the 200 replicates. We also scaled ${\Delta \text{G}}_{\text{true}}$ by setting ${\Delta \text{G}}_{\text{true}}$ realised by POCS to 100 in each combination of breeding scheme and prediction method.

${\Delta \text{G}}_{\text{true}}$ in each replicate was calculated as a linear regression of ${\text{G}}_{t}$ on $t$, where ${\text{G}}_{t}$ is the average TBV of animals born in generations $t$ = 4 … 10. ${\Delta \text{G}}_{\text{true}}$ was presented as a linear regression because ${\text{G}}_{t}$ was linear over $t$. ${\Delta \text{F}}_{\text{true}}$ in each replicate was calculated as $1 - { \exp }\left({\beta} \right)$, where ${\beta}$ is a linear regression of ${ \ln }\left({1 - {\text{F}}_{t}} \right)$ on $t$, and ${\text{F}}_{t}$ is the average coefficient of true inbreeding for animals born in generations $t$ = 4 … 10 [12, 13]. These transformations were made because ${ \ln }\left({1 - {\text{F}}_{t}} \right)$, not ${\text{F}}_{t}$, was linear over $t$. ${\text{F}}_{t}$ was calculated as ${\text{F}}_{t} = \frac{1}{{n_{t} n_{\text{IBD}}}}\mathop \sum \nolimits_{i = 1}^{{n_{t}}} \mathop \sum \nolimits_{j = 1}^{{n_{\text{IBD}}}} {\delta}_{ij}$, where $n_{t}$ is the number of animals born in generation $t$, $n_{\text{IBD}}$ = 6012 is the number of IBD loci, and ${\delta}_{ij}$ is the IBD status of animal $i$ $\left({i = 1 \ldots n_{t}} \right)$ at IBD locus $j$ $\left({j = 1 \ldots n_{\text{IBD}}} \right)$. ${\delta}_{ij}$ was equal to 1 if animal $i$ was homozygous at IBD locus $j$, and 0 otherwise.

### Rates of pedigree and genomic inbreeding

We present rates of pedigree inbreeding realised by POCS and rates of genomic inbreeding realised by GOCS at $0.01 {\Delta \text{F}}_{\text{true}}$ in each combination of breeding scheme and prediction method. Rates of pedigree and genomic inbreeding were calculated as for ${\Delta \text{F}}_{\text{true}}$ with ${\text{F}}_{t}$ replaced by average coefficients of pedigree and genomic inbreeding for animals born in generations $t$ = 4 … 10. The coefficient of genomic inbreeding for animal $i$ was calculated as ${\text{G}}_{ii} {-}1.0$, where ${\text{G}}_{ii}$ is the $i$th diagonal element of ${\mathbf{G}}$ used in GOCS.

### Mechanisms underlying POCS and GOCS

We present results that highlight the mechanisms underlying POCS and GOCS. These results are only presented for POCS and GOCS with PBLUP and GBLUP in breeding scheme M25L5; results from breeding schemes M100L5 and M25L20 were similar to those from breeding scheme M25L5. Two of the results—response frontiers and minimum ${\Delta \text{F}}_{\text{true}}$—involved additional simulations. All of these results are presented as means (± SD) of 200 replicates.

#### Changes in allele frequencies at markers and QTL

We present the average absolute changes in allele frequencies at markers and QTL at $0.01 {\Delta \text{F}}_{\text{true}}$ and the average increase in the frequencies of favourable alleles at the QTL. Changes in allele frequencies were calculated from generations $t$ = 4 to $t$ = 10 using animals born in generations $t$ = 4 and $t$ = 10. The frequency changes in each replicate were averaged over the 54,218 markers and 7702 QTL.

#### Variance in rate of identity-by-descent

We present the variance in rate of IBD between the 6012 IBD loci at $0.01 {\Delta \text{F}}_{\text{true}}$. Rate of IBD at each locus in each replicate was calculated as $1 - { \exp }\left({\beta} \right)$, where ${\beta}$ is a linear regression of ${ \ln }\left({1 - {\text{F}}_{it}} \right)$ on $t$, and ${\text{F}}_{it}$ is the proportion of animals born in generations $t$ = 4 … 10 that were IBD at locus $i$ $\left({i = 1 \ldots 6012} \right)$.

#### Numbers of candidates and families that were allocated matings

We present the number of male candidates that were allocated matings at $0.01 {\Delta \text{F}}_{\text{true}}$ and the numbers of half and full-sib families with male or female candidates that were allocated matings. The numbers in each replicate were averaged over generations $t$ = 4 … 10. The number of female candidates that were allocated matings was not presented because 25 females were always allocated a single mating in breeding scheme M25L5.

#### Rank and rank deviations

We present the average ranks and average-rank deviations of males and females that were allocated matings within full-sib families at $0.01 {\Delta \text{F}}_{\text{true}}$ when males and females within each full-sib family were ranked by predicted breeding value. The average rank of males that were allocated matings in each full-sib family was calculated when males were ranked from 1 … $n_{{{\text{male}}i}}$, where $n_{{{\text{male}}i}}$ is the number of males in the $i$th full-sib family. The average-rank deviation of males in each full-sib family was calculated as the difference between their average rank and their average-minimum rank, where average-minimum rank is the average rank had those males that were allocated matings been the highest-ranked males in their full-sib families. The average ranks and average-rank deviations in each generation were averaged across full-sib families with males that were allocated matings. The generation averages in each replicate were averaged over generations $t$ = 4 … 10. The average rank and average-rank deviation of females were calculated as for males.

#### Response frontiers

We present response frontiers for POCS and GOCS with PBLUP and GBLUP: ${\Delta \text{G}}_{\text{true}}$ plotted against ${\Delta \text{F}}_{\text{true}}$. Different ${\Delta \text{F}}_{\text{true}}$ were realised by applying different penalties, $\omega$, in Eq. (1). Response frontiers tell us if the relative ${\Delta \text{G}}_{\text{true}}$ realised by POCS and GOCS at $0.01 {\Delta \text{F}}_{\text{true}}$ is also realised across a range of ${\Delta \text{F}}_{\text{true}}$.

#### Minimum rates of true inbreeding

We present the minimum ${\Delta \text{F}}_{\text{true}}$ realised by POCS and GOCS when we relaxed selection for predicted breeding value. POCS was carried out as described previously with the exception that Eq. (1) was reduced to ${\text{U}}_{t} \left({\mathbf{c}} \right) = - {\mathbf{c}}^{\prime}{\mathbf{Ac}}$ for POCS. With GOCS, ${\mathbf{A}}$ was replaced by ${\mathbf{G}}$. Minimum ${\Delta \text{F}}_{\text{true}}$ provides insight into the effectiveness of POCS and GOCS to control ${\Delta \text{F}}_{\text{true}}$.

### Software

The simulations were run using the program ADAM [14]. PBLUP and GBLUP were predicted using DMU version 6 [15]. OCS was carried out by EVA [16].

## Results

### Rates of true genetic gain

POCS realised more ${\Delta \text{G}}_{\text{true}}$ than GOCS at $0.01 {\Delta \text{F}}_{\text{true}}$ in all combinations of breeding scheme and prediction method. POCS realised 14 to 33% more ${\Delta \text{G}}_{\text{true}}$ than GOCS with PBLUP in our three breeding schemes (Table 1). It realised 1.5 to 5.7% more ${\Delta \text{G}}_{\text{true}}$ than GOCS with GBLUP and 0.3 to 1.4% more ${\Delta \text{G}}_{\text{true}}$ than GOCS with our reference prediction, TBV.

**Table 1:** Rate of true genetic gain realised by POCS, GOCS, and IOCS at $0.01\varvec{ }{\Delta F}_{{\text{true}}}$ in three breeding schemes with three predictions methods (PBLUP, GBLUP, and TBV)

Prediction	Scheme	OCS	${\Delta \text{G}}_{\text{true}}$	${\Delta \text{G}}_{\text{scaled}}$	${\Delta \text{F}}_{\text{pedigree}}$	${\Delta \text{F}}_{\text{genomic}}$
PBLUP	M25L5	POCS	0.379	100.0	0.0098	
		GOCS	0.317	83.6		0.0071
		IOCS	0.356	93.9		
	M25L20	POCS	0.570	100.0	0.0094	
		GOCS	0.429	75.3		0.0061
		IOCS	0.544	95.4		
	M100L5	POCS	0.554	100.0	0.0094	
		GOCS	0.485	87.5		0.0080
		IOCS	0.534	96.4		
GBLUP	M25L5	POCS	0.398	100.0	0.0088	
		GOCS	0.390	98.0		0.0099
		IOCS	0.409	102.8		
	M25L20	POCS	0.703	100.0	0.0074	
		GOCS	0.665	94.6		0.0117
		IOCS	0.720	102.4		
	M100L5	POCS	0.658	100.0	0.0076	
		GOCS	0.648	98.5		0.0127
		IOCS	0.670	101.8		
TBV	M25L5	POCS	0.773	100.0	0.0090	
		GOCS	0.762	98.6		0.0101
		IOCS	0.782	101.2		
	M25L20	POCS	1.149	100.0	0.0079	
		GOCS	1.143	99.5		0.0131
		IOCS	1.162	101.1		
	M100L5	POCS	0.999	100.0	0.0080	
		GOCS	0.996	99.7		0.0124
		IOCS	1.012	101.3		

Rates of absolute and scaled true genetic gain (${\Delta \text{G}}_{\text{true}}$ and ${\Delta \text{G}}_{\text{scaled}}$), rates of pedigree inbreeding (${\Delta \text{F}}_{\text{pedigree}}$) realised by POCS, and rates of genomic inbreeding (${\Delta \text{F}}_{\text{genomic}}$) realised by GOCS are means of 200 simulation replicates. ${\Delta \text{G}}_{\text{scaled}}$ was calculated by setting ${\Delta \text{G}}_{\text{true}}$ realised by POCS to 100 in each combination of breeding scheme and prediction method. SD between the replicates ranged from 0.00114 to 0.00256 (${\Delta \text{F}}_{\text{true}}$), 0.0288 to 0.0559 (${\Delta \text{G}}_{\text{true}}$), 2.51 to 14.45 (${\Delta \text{G}}_{\text{scaled}}$), 0.00743 to 0.00979 (${\Delta \text{F}}_{\text{pedigree}}$), and 0.00153 to 0.00270 (${\Delta \text{F}}_{\text{genomic}}$)

POCS also realised more ${\Delta \text{G}}_{\text{true}}$ than IOCS at $0.01 {\Delta \text{F}}_{\text{true}}$ with PBLUP. With PBLUP, POCS realised 3.7 to 6.5% more ${\Delta \text{G}}_{\text{true}}$ than IOCS in our three breeding schemes (Table 1). In turn, IOCS realised 10 to 27% more ${\Delta \text{G}}_{\text{true}}$ than GOCS. While POCS realised more ${\Delta \text{G}}_{\text{true}}$ than IOCS with PBLUP at $0.01 {\Delta \text{F}}_{\text{true}}$, IOCS realised a little more ${\Delta \text{G}}_{\text{true}}$ than POCS with GBLUP and TBV. With GBLUP and TBV, IOCS realised 1.8 to 2.8% and 1.1 to 1.3% more ${\Delta \text{G}}_{\text{true}}$ than POCS. IOCS realised 3.4 to 8.3% and 1.6 to 2.6% more ${\Delta \text{G}}_{\text{true}}$ than GOCS with GBLUP and TBV.

### Rates of pedigree and genomic inbreeding

Pedigree relationships used by POCS underestimated $0.01 {\Delta \text{F}}_{\text{true}}$ in all combinations of breeding scheme and prediction method. POCS underestimated $0.01 {\Delta \text{F}}_{\text{true}}$ by 2 to 6% with PBLUP in our three breeding schemes (Table 1). With GBLUP and TBV, it underestimated $0.01 {\Delta \text{F}}_{\text{true}}$ by 10 to 26%. By contrast, genomic relationships used by GOCS underestimated $0.01 {\Delta \text{F}}_{\text{true}}$ by 20 to 39% with PBLUP, but overestimated $0.01 {\Delta \text{F}}_{\text{true}}$ by as much as 31% with GBLUP and TBV.

The following sections present results that highlight the mechanisms underlying POCS and GOCS. The results are presented for breeding scheme M25L5 with PBLUP and GBLUP.

### Changes in allele frequencies at markers and QTL

POCS generated larger changes in allele frequencies at markers and QTL than GOCS at $0.01 {\Delta \text{F}}_{\text{true}}$. In breeding scheme M25L5 with PBLUP and GBLUP, the average absolute changes in allele frequencies generated by POCS at markers and QTL were about 4% larger than the changes generated by GOCS (Table 2). By contrast, POCS increased the average frequency of favourable alleles at QTL by 20% more than GOCS with PBLUP and by 4.8% more than GOCS with GBLUP.

**Table 2:** Average absolute changes in allele frequencies at markers and QTL, and average increase in the frequencies of favourable QTL alleles generated by POCS and GOCS at $0.01\varvec{ }{\Delta F}_{{\text{true}}}$ in breeding scheme M25L5 with two prediction methods (PBLUP and GBLUP)

Prediction	OCS	Absolute-marker alleles	Absolute-QTL alleles	Favourable-QTL alleles
PBLUP	POCS	0.0475	0.0472	0.00428
	GOCS	0.0456	0.0455	0.00356
GBLUP	POCS	0.0493	0.0487	0.00459
	GOCS	0.0471	0.0468	0.00438

Changes in allele frequencies are means of 200 simulation replicates. SD between the replicates ranged from 0.00132 to 0.00159 (absolute-marker alleles and absolute-QTL alleles) and from 0.00078 to 0.00085 (favourable QTL alleles)

### Variance in rate of identity-by-descent

POCS and GOCS generated similar variances in rate of IBD between the 6012 IBD loci at $0.01 {\Delta \text{F}}_{\text{true}}$. This was highlighted by breeding scheme M25L5 with PBLUP and GBLUP (Table 3).

**Table 3:** Variance in rate of IBD between 6012 IBD loci generated by POCS and GOCS at $0.01\varvec{ }{\Delta F}_{{\text{true}}}$ in breeding scheme M25L5 with two prediction methods (PBLUP and GBLUP)

Prediction	OCS	Variance
PBLUP	POCS	4.52 × 10^−5^
	GOCS	4.56 × 10^−5^
GBLUP	POCS	4.48 × 10^−5^
	GOCS	4.64 × 10^−5^

Variances are means of 200 simulation replicates. SD between the replicates ranged from 5.607 $\times$ 10^−6^ to 7.687 $\times$ 10^−6^

### Numbers of candidates and families that were allocated matings

#### Males

POCS allocated matings to more male candidates than GOCS at $0.01 {\Delta \text{F}}_{\text{true}}$. In breeding scheme M25L5 with PBLUP, POCS allocated matings to 10.1% more male candidates than GOCS (Table 4). With GBLUP, POCS allocated matings to 5.9% more male candidates.

**Table 4:** Number of male candidates allocated matings, and numbers of half and full-sib families with candidates allocated matings by POCS and GOCS at $0.01\varvec{ }{\Delta F}_{{\text{true}}}$ in breeding scheme M25L5 with two prediction methods (PBLUP and GBLUP)

Prediction	OCS	Males	Half-sibs	Full-sibs
PBLUP	POCS	19.7	17.8	21.8
	GOCS	17.9	16.1	21.3
GBLUP	POCS	19.9	18.9	23.0
	GOCS	18.8	17.1	21.9

Numbers are means of 200 simulation replicates. SD between the replicates ranged from 0.72 to 0.87 (males), 0.79 to 0.93 (half-sibs), and 0.50 to 0.60 (full-sibs)

#### Half and full-sib families

Selection candidates that were allocated matings by POCS were from more half and full-sib families than GOCS at $0.01 {\Delta \text{F}}_{\text{true}}$. In breeding scheme M25L5 with PBLUP, POCS allocated matings to candidates from 10.6% more half-sib and 2.3% more full-sib families than GOCS (Table 4). With GBLUP, POCS allocated matings to candidates from 10.5 and 5.0% more half and full-sib families.

### Rank and rank deviations

POCS allocated matings to higher-ranked candidates within full-sib families than GOCS at $0.01 {\Delta \text{F}}_{\text{true}}$. In breeding scheme M25L5 with PBLUP and GBLUP, the average ranks of males and females that were allocated matings by POCS were 8.5 to 10.8% lower than those allocated matings by GOCS (Table 5). Not only did POCS allocate matings to higher-ranked candidates within full-sib families, candidates that were allocated matings by POCS were always the highest-ranked males and females in their full-sib families. The average ranks of the males and females that were allocated matings by POCS did not deviate from their average-minimum ranks—their average-rank deviations were zero. With GOCS, the average ranks of the males and females deviated from their average-minimum ranks by about 10%.

**Table 5:** Average ranks and average-rank deviations of males and females allocated matings by POCS and GOCS at $0.01\varvec{ }{\Delta F}_{{\text{true}}}$ in breeding scheme M25L5 with two prediction methods (PBLUP and GBLUP)

Prediction	OCS	Rank_Males_	Deviation_Males_	Rank_Females_	Deviation_Females_
PBLUP	POCS	1.18 ± 0.025	0	1.26 ± 0.026	0
	GOCS	1.29 ± 0.056	0.13 ± 0.044	1.39 ± 0.044	0.12 ± 0.035
GBLUP	POCS	1.16 ± 0.022	0	1.24 ± 0.026	0
	GOCS	1.30 ± 0.052	0.13 ± 0.044	1.39 ± 0.043	0.13 ± 0.038

Average ranks and average-rank deviations are means (± SD) of 200 simulation replicates

### Response frontiers

**Fig. 2:**
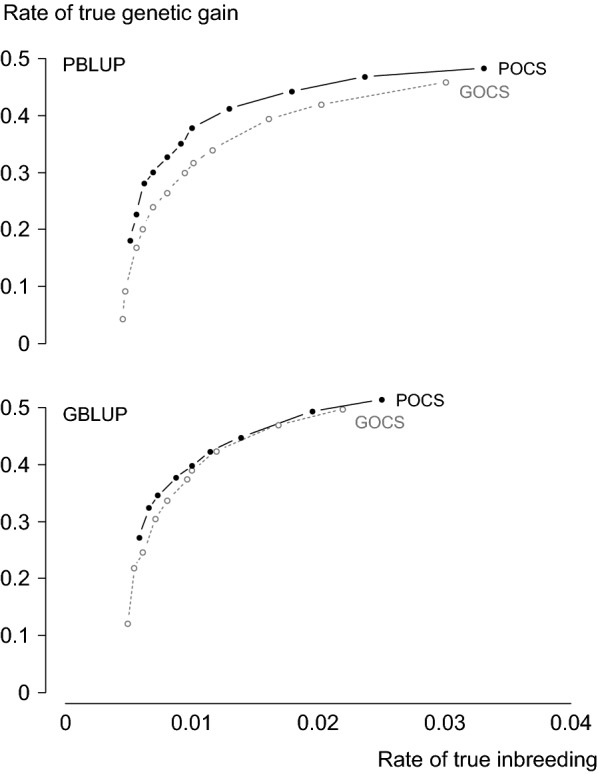
Rate of true genetic gain realised by POCS and GOCS plotted against ${\Delta \text{F}}_{\text{true}}$ in breeding scheme M25L5 with two prediction methods (PBLUP and GBLUP)

There were two main features of our response frontiers. First, POCS continued to realise more ${\Delta \text{G}}_{\text{true}}$ than GOCS across a range of ${\Delta \text{F}}_{\text{true}}$. Second, both POCS and GOCS realised less ${\Delta \text{G}}_{\text{true}}$ as ${\Delta \text{F}}_{\text{true}}$ decreased, but ${\Delta \text{G}}_{\text{true}}$ realised by POCS decreased at a slower rate than GOCS. These two features are illustrated by the response frontiers for POCS and GOCS with PBLUP and GBLUP in breeding scheme M25L5 (Fig. 2). With PBLUP, POCS realised about 6% more ${\Delta \text{G}}_{\text{true}}$ than GOCS at $0.03 {\Delta \text{F}}_{\text{true}}$, 20% more ${\Delta \text{G}}_{\text{true}}$ at $0.01 {\Delta \text{F}}_{\text{true}}$, and 40% more ${\Delta \text{G}}_{\text{true}}$ at $0.006 {\Delta \text{F}}_{\text{true}}$. With GBLUP, POCS and GOCS realised similar ${\Delta \text{G}}_{\text{true}}$ at ${\Delta \text{F}}_{\text{true}}$ higher than about 0.015. POCS realised 2% more ${\Delta \text{G}}_{\text{true}}$ than GOCS at $0.01 {\Delta \text{F}}_{\text{true}}$ and almost 10% more ${\Delta \text{G}}_{\text{true}}$ at about $0.006 {\Delta \text{F}}_{\text{true}}$.

### Minimum rates of true inbreeding

POCS realised higher minimum ${\Delta \text{F}}_{\text{true}}$ than GOCS when we relaxed selection for predicted breeding value. In breeding scheme M25L5, the minimum ${\Delta \text{F}}_{\text{true}}$ realised by POCS was 6.1% higher than the minimum ${\Delta \text{F}}_{\text{true}}$ realised by GOCS (mean ± SD of 200 replicates: 0.0050 ± 0.00147 *vs* 0.0047 ± 0.00144).

## Discussion

Our findings supported our premise that POCS realises at least as much ${\Delta \text{G}}_{\text{true}}$ as GOCS at the same ${\Delta \text{F}}_{\text{true}}$. When we calibrated POCS and GOCS to $0.01 {\Delta \text{F}}_{\text{true}}$, we found that POCS always realised more ${\Delta \text{G}}_{\text{true}}$ than GOCS, regardless of the breeding scheme that we simulated or the information used to predict breeding values. This makes POCS an attractive method of OCS to use in breeding schemes, including schemes that use GBLUP. It also highlights that the potential for GOCS to trace changes in allele frequencies at markers does not guarantee more ${\Delta \text{G}}_{\text{true}}$. Our findings are supported by the results of Sonesson et al. [4] and Clark et al. [5], but not the recommendation by Sonesson et al. [4], who reasoned that the same information used to predict ${\Delta \text{G}}_{\text{true}}$ should also be used to predict and control ${\Delta \text{F}}_{\text{true}}$. It would be worthwhile reassessing the recommendation by Sonesson et al. [4], given that their reasoning did not consider ${\Delta \text{G}}_{\text{true}}$ and their study made GOCS the method-of-choice for OCS with GBLUP. While we predicted that POCS would realise at least as much ${\Delta \text{G}}_{\text{true}}$ as GOCS, we were surprised to find that our reference OCS, IOCS, realised only marginally more ${\Delta \text{G}}_{\text{true}}$ than POCS with GBLUP and TBV. This result provides little incentive to use GOCS and highlights that we have more to learn before we can control inbreeding using genomic relationships in selective-breeding schemes. Until we can do so, POCS remains a worthy method of OCS because it realises more ${\Delta \text{G}}_{\text{true}}$ than GOCS at the same ${\Delta \text{F}}_{\text{true}}$.

POCS realised more ${\Delta \text{G}}_{\text{true}}$ than GOCS because it managed expected genetic drift without restricting selection at QTL. It did this by applying a penalty to ${\mathbf{c}}^{\prime}{\mathbf{Ac}}$. ${\mathbf{c}}^{\prime}{\mathbf{Ac}}$ can be rewritten as ${\mathbf{c^{\prime}Ac}} = {\mathbf{c}}^{\prime}{\mathbf{LL}}^{\prime}{\mathbf{c}}$, where ${\mathbf{L}}$ is a normed lower-triangular matrix that describes the expected genetic contribution that an ancestor makes to its descendants, and ${\mathbf{L}}^{\prime}{\mathbf{c}}$ is a vector of expected genetic contributions from candidates and ancestors to the next generation [17, 18]. Penalising ${\mathbf{c^{\prime}Ac}} = {\mathbf{c}}^{\prime}{\mathbf{LL}}^{\prime}{\mathbf{c}}$ penalised increases in expected genetic contributions quadratically, where the sum of squares of expected contributions is a function of expected genetic drift [19]. Managing expected genetic drift managed the variance in changes in allele frequencies at hypothetical neutral loci. These neutral loci are assumed to be unlinked to QTL alleles [3]. Because they were unlinked, POCS allowed the frequencies of favourable alleles at QTL to be increased by selection. By contrast, GOCS penalised changes in allele frequencies at markers that were generated by genetic drift and selection. It applied a penalty to ${\mathbf{c}}^{\prime}{\mathbf{Gc}} = {\mathbf{c}}^{\prime}{\mathbf{ZZ}}^{\prime}{\mathbf{c}}/{\text{s}}$, where ${\mathbf{Z}}^{\prime}{\mathbf{c}}/2$ is a vector of changes in allele frequency at each marker [3] and these changes were measured as deviations from allele frequencies in the base populations. Penalising ${\mathbf{c^{\prime}Gc}} = {\mathbf{c^{\prime}ZZ^{\prime}c}}/{\text{s}}$ penalised changes in allele frequencies at markers quadratically; markers with the largest frequency changes were penalised hardest. Because these marker alleles were in LD with QTL alleles, GOCS restricted changes in allele frequencies at QTL. This explanation highlights the problem with GOCS in its current form: it penalises changes in allele frequencies at all markers when, in fact, we need to change allele frequencies at some markers to increase the frequencies of favourable alleles at QTL. So, by managing expected genetic drift, POCS realises more ${\Delta \text{G}}_{\text{true}}$ than GOCS at the same ${\Delta \text{F}}_{\text{true}}$ because it allows selection to increase the frequencies of favourable alleles at QTL more than GOCS.

Deductive reasoning tells us that POCS also generated different IBD profiles across the genome than GOCS at $0.01 {\Delta \text{F}}_{\text{true}}$. POCS must have generated more IBD than GOCS in regions of the genome that harboured QTL and less IBD in regions that lacked QTL, given that (1) POCS realised more ${\Delta \text{G}}_{\text{true}}$ than GOCS, (2) POCS generated larger increases in the frequencies of favourable alleles at QTL, (3) QTL alleles were in LD with IBD alleles, (4) areas under IBD profiles increase at the same rate at the same ${\Delta \text{F}}_{\text{true}}$, and (5) POCS and GOCS generated similar variances in rate of IBD between IBD loci. While the IBD generated by POCS was associated with QTL location, the IBD generated by GOCS must have been associated with both QTL location and marker density because the markers used by GOCS to predict and control ${\Delta \text{F}}_{\text{true}}$ were randomly distributed across the genome. GOCS presumably generated most IBD in regions of the genome that harboured QTL with low marker densities, least IBD in regions that lacked QTL with high marker densities, and intermediate IBD in other regions of the genome. We did not present IBD profiles for POCS and GOCS because we simulated many QTL, each with a small change in allele frequency. This resulted in differences in IBD between POC and GOCS at each IBD locus that were small and difficult to detect visually. It would be worthwhile carrying out simulations to test unequivocally that POCS generates more IBD than GOCS in regions of the genome that harbour QTL. Increasing the frequencies of favourable alleles at QTL while restricting most of the IBD to regions of the genome that harbour these QTL is, after all, how we want to realise ${\Delta \text{G}}_{\text{true}}$ at acceptable ${\Delta \text{F}}_{\text{true}}$ in animal breeding. Therefore, not only does POCS allow selection to increase the frequencies of favourable alleles at QTL more than GOCS at the same ${\Delta \text{F}}_{\text{true}}$, it is probably also more aligned with the objectives of animal breeding by restricting most IBD to regions of the genome that harbour QTL.

A direct consequence of managing expected genetic drift was that POCS allocated matings to different selection candidates than GOCS. There were two major differences. First, POCS allocated matings to more candidates from more half and full-sib families than GOCS to realise $0.01 {\Delta \text{F}}_{\text{true}}$. POCS did this because it could neither differentiate between pairwise relationships within full-sib families—they had the same expected relationships—nor could it trace increases in allele frequencies at IBD loci that were in regions of the genome under the influence of genetic drift and selection. These regions were prone to higher ${\Delta \text{F}}_{\text{true}}$ than predicted by pedigree relationships. Candidates that were allocated matings by POCS tended to share more QTL alleles, more genomic regions flanking the QTL, and more IBD alleles than predicted. POCS compensated for this by allocating matings at rates of pedigree inbreeding that were lower than $0.01 {\Delta \text{F}}_{\text{true}}$. It used variation in expected relationships between families to allocate matings to more candidates from more families. By contrast, GOCS allocated matings to fewer candidates from fewer families than POCS because it could differentiate between pairwise relationships and exploit some of the variation in IBD relationships within full-sib families. Realising the same ${\Delta \text{F}}_{\text{true}}$ with fewer breeding animals made GOCS a more effective control of ${\Delta \text{F}}_{\text{true}}$ than POCS and shows that GOCS does provide valuable information for inbreeding control—it was just not as effective at realising ${\Delta \text{G}}_{\text{true}}$. Further evidence that GOCS controlled ${\Delta \text{F}}_{\text{true}}$ more effectively was that it realised lower minimum ${\Delta \text{F}}_{\text{true}}$ than POCS when we relaxed selection for predicted breeding value. With no selection for predicted breeding value, the objective was to restrict increases in the frequencies of IBD alleles, which GOCS did more effectively than POCS. But despite GOCS being a more effective control of ${\Delta \text{F}}_{\text{true}}$, POCS still realised a minimum ${\Delta \text{F}}_{\text{true}}$ of 0.005 with only 25 matings per generation in breeding scheme M25L5. This was well within the 0.005–0.01 range of ${\Delta \text{F}}_{\text{true}}$ that is considered acceptable for breeding schemes [20]. Therefore, POCS should still be able to realise ${\Delta \text{F}}_{\text{true}}$ that is considered acceptable in most breeding schemes by allocating matings to more candidates from more full and half-sib families.

The second difference was that POCS allocated matings to higher-ranked candidates within full-sib families than GOCS at $0.01 {\Delta \text{F}}_{\text{true}}$. Candidates that were allocated matings by POCS were always the highest-ranked males and females in their full-sib families. Allocating matings to the highest-ranked candidates generated the extra ${\Delta \text{G}}_{\text{true}}$ realised by POCS as these candidates tended to share favourable alleles at QTL. POCS allowed matings to be allocated to these candidates because all full-sibs have the same pairwise relationships based on pedigree relationships; candidates from the same full-sib family incurred the same penalty regardless of rank. On the other hand, GOCS could not always allocate matings to the highest-ranked candidates. Just as these candidates tended to share QTL alleles, they also shared marker alleles. Allocating matings to them was penalised by GOCS because it generated larger changes in allele frequencies at markers. So, POCS more than compensated for allocating matings to more candidates from more half and full-sib families to realise the same ${\Delta \text{F}}_{\text{true}}$ as GOCS. It allocated these matings to higher-ranked candidates within full-sib families, which increased the frequencies of favourable alleles at QTL and realised more ${\Delta \text{G}}_{\text{true}}$.

Not only did POCS realise more ${\Delta \text{G}}_{\text{true}}$ than GOCS at $0.01 {\Delta \text{F}}_{\text{true}}$, it also realised more ${\Delta \text{G}}_{\text{true}}$ across a range of ${\Delta \text{F}}_{\text{true}}$. At ${\Delta \text{F}}_{\text{true}}$ higher than $0.01 {\Delta \text{F}}_{\text{true}}$, POCS and GOCS realised similar ${\Delta \text{G}}_{\text{true}}$. Most selection emphasis was on ${\Delta \text{G}}_{\text{true}}$, and both POCS and GOCS tended to allocate matings to the same highly-ranked candidates that would have been allocated matings by truncation selection. At lower ${\Delta \text{F}}_{\text{true}}$, the mechanisms that differentiate POCS from GOCS became more pronounced and POCS realised relatively more ${\Delta \text{G}}_{\text{true}}$ than GOCS. POCS allocated matings to even more candidates from more full and half-sib families to reduce ${\Delta \text{F}}_{\text{true}}$. Candidates that were allocated matings by POCS continued to be the highest-ranked males and females in their full-sib families and the frequencies of favourable alleles at QTL continued to increase, albeit at slower rates. On the other hand, GOCS penalised changes in allele frequencies at markers even harder at lower ${\Delta \text{F}}_{\text{true}}$. Candidates that were allocated matings by GOCS differed more for predicted IBD relationships and they were less likely to be the highest-ranked males and females in their full-sib families. This further restricted changes in allele frequencies at QTL. That is, penalising changes in allele frequencies at markers imposes increasingly larger restrictions on changes in allele frequencies at QTL at lower ${\Delta \text{F}}_{\text{true}}$ than penalising increases in expected genetic contributions. Therefore, the mechanisms that underlie POCS and GOCS apply across a range of ${\Delta \text{F}}_{\text{true}}$ with POCS realising relatively more ${\Delta \text{G}}_{\text{true}}$ than GOCS at lower ${\Delta \text{F}}_{\text{true}}$ because it allows relatively larger changes in the frequencies of favourable alleles at QTL.

Pedigree and genomic relationships used by POCS and GOCS were only predictors of ${\Delta \text{F}}_{\text{true}}$. Pedigree relationships used by POCS always underestimated $0.01 {\Delta \text{F}}_{\text{true}}$ when we selected for predicted breeding value because they could not trace increases in allele frequencies at IBD loci that were in regions of the genome under the influence of selection. Genomic relationships used by GOCS did not predict ${\Delta \text{F}}_{\text{true}}$ more accurately than pedigree relationships, even though GOCS controlled ${\Delta \text{F}}_{\text{true}}$ more effectively than POCS. GOCS underestimated $0.01 {\Delta \text{F}}_{\text{true}}$ with PBLUP, but overestimated $0.01 {\Delta \text{F}}_{\text{true}}$ with GBLUP and TBV. There are two reasons why GOCS did not predict ${\Delta \text{F}}_{\text{true}}$ more accurately than POCS. First, the marker alleles used to predict ${\Delta \text{F}}_{\text{true}}$ were not in complete LD with IBD alleles. Second, the markers were randomly distributed across the genome. Random distribution implies uneven control of IBD across the genome because GOCS could only control inbreeding using marker alleles. GOCS had to increase inbreeding control in regions of the genome with high marker densities to compensate for reduced inbreeding control in regions with low marker densities. Observable and accurate predictors of ${\Delta \text{F}}_{\text{true}}$ are central to breeding schemes for two reasons. First, they provide a measure of risk; the risk of breeding schemes being adversely impacted by inbreeding depression and loss of genetic variation [3]. Second, they enable OCS to increase selection differentials by allocating matings to selection candidates that realise predicted rates of inbreeding that are close to desired ${\Delta \text{F}}_{\text{true}}$. With no obvious relationship between ${\Delta \text{F}}_{\text{true}}$ and rates of pedigree and genomic inbreeding, we are unable to calibrate POCS and GOCS to realise the desired ${\Delta \text{F}}_{\text{true}}$. This makes inbreeding control using POCS or GOCS challenging. Clearly, observable and accurate predictors of ${\Delta \text{F}}_{\text{true}}$ are needed to better manage risk and increase selection differentials in animal breeding.

Even with accurate predictors of ${\Delta \text{F}}_{\text{true}}$, GOCS in its current form is still unlikely to realise more ${\Delta \text{G}}_{\text{true}}$ than POCS at the same ${\Delta \text{F}}_{\text{true}}$. In other words, accurate prediction of ${\Delta \text{F}}_{\text{true}}$ is not enough to maximise ${\Delta \text{G}}_{\text{true}}$ at the same ${\Delta \text{F}}_{\text{true}}$. The reason is that prediction of ${\Delta \text{F}}_{\text{true}}$ and inbreeding control are different concepts when maximising ${\Delta \text{G}}_{\text{true}}$ at the same ${\Delta \text{F}}_{\text{true}}$. This was highlighted by our reference OCS, IOCS. IOCS realised more ${\Delta \text{G}}_{\text{true}}$ than GOCS at $0.01 {\Delta \text{F}}_{\text{true}}$ because it had perfect knowledge of ${\Delta \text{F}}_{\text{true}}$. It controlled ${\Delta \text{F}}_{\text{true}}$ with the same IBD alleles that were used to calculate ${\Delta \text{F}}_{\text{true}}$. This suggests that GOCS will realise more ${\Delta \text{G}}_{\text{true}}$ if genomic relationships could be used to predict ${\Delta \text{F}}_{\text{true}}$ more accurately. However, the amount of extra ${\Delta \text{G}}_{\text{true}}$ is unlikely to result in GOCS realising more ${\Delta \text{G}}_{\text{true}}$ than POCS, given that IOCS, at best, only realised marginally more ${\Delta \text{G}}_{\text{true}}$ than POCS at $0.01 {\Delta \text{F}}_{\text{true}}$. IOCS realised only marginally more ${\Delta \text{G}}_{\text{true}}$ than POCS because it penalised increases in allele frequencies at IBD loci in the same way that GOCS penalised changes in allele frequencies at markers. It applied a penalty to ${\mathbf{c}}^{\prime}{\mathbf{Bc}} = {\mathbf{c}}^{\prime}{\mathbf{DD}}^{\prime}{\mathbf{c}}$, where ${\mathbf{D}}$ is a matrix of counts of each unique allele at each IBD locus that was inherited by each animal, and ${\mathbf{D}}^{\prime}{\mathbf{c}}$ is a vector of the numbers of each allele at each IBD locus that were expected to be passed on to the next generation. Penalising ${\mathbf{c}}^{\prime}{\mathbf{Bc}} = {\mathbf{c}}^{\prime}{\mathbf{DD}}^{\prime}{\mathbf{c}}$ penalised increases in the expected numbers of IBD alleles quadratically. This presumably generated flat IBD profiles across the genome and restricted changes in allele frequencies at QTL. Like GOCS, IOCS needed to increase the frequencies of some IBD alleles to increase the frequencies of favourable alleles at QTL. So, GOCS in its current form, where changes in allele frequencies at all markers are penalised, is unlikely to ever realise more ${\Delta \text{G}}_{\text{true}}$ than POCS at the same ${\Delta \text{F}}_{\text{true}}$.

If GOCS is to realise more ${\Delta \text{G}}_{\text{true}}$ than POCS at the same ${\Delta \text{F}}_{\text{true}}$, we will need to change the way that genomic relationships are used to control ${\Delta \text{F}}_{\text{true}}$. Rather than penalise changes in allele frequencies at all markers, we should probably allow changes in allele frequencies at some markers by varying the level of inbreeding control and rate of IBD across the genome while controlling ${\Delta \text{F}}_{\text{true}}$ at acceptable levels. This will involve relaxing inbreeding control in regions of the genome that harbour QTL, allowing selection to increase the frequencies of favourable alleles at QTL. At the same time, we will need to increase inbreeding control to reduce genetic drift in regions of the genome that lack QTL. Varying the level of inbreeding control across the genome could be carried out in GOCS by constructing genomic-relationship matrices that weight markers in regions of the genome that harbour QTL lower than markers in regions that lack QTL. Weighted genomic-relationship matrices have been used in genomic prediction [21, 22]. An alternative approach is to construct genomic-relationship matrices by fixing the frequencies of mutant alleles at markers, ${\mathbf{p}}$, to desired frequencies rather than frequencies in base populations. This approach would cause GOCS to penalise deviations from the desired allele frequencies. While these approaches are simple in theory, implementing them in practice requires that we overcome a major hurdle: we do not know where many, if any, of the QTL are located on the genome. We do not know what we want to change and in what direction, nor do we know which regions of the genome can tolerate being IBD. Overcoming this hurdle will require biological information about the QTL that control traits under selection, traits that might be under selection in future, and unobserved fitness traits. Unfortunately, this information is unlikely to become available soon. Without it, there is no guarantee that GOCS will realise more ${\Delta \text{G}}_{\text{true}}$ or that it will control IBD in regions of the genome that are susceptible to IBD. Therefore, GOCS should realise more ΔG_true_ than POCS at the same ΔF_true_ when we relax inbreeding control in regions of the genome that harbour QTL, but implementing this in practice will require biological information about QTL.
